# Bridging the stroke care gap: development and validation of CaknaStrok Education Package (CEP) for caregivers of stroke survivors in Malaysia

**DOI:** 10.1136/bmjopen-2025-111268

**Published:** 2026-02-26

**Authors:** Norsima Nazifah Sidek, Sureshkumar Kamalakannan, Kamarul Imran Musa, Tuan Siti Mastazliha Long Tuan Kechik, Nurfaten Hamzah, Rose Izura Abd Hamid, Daryani Darus, Khairul Azmi Ibrahim, Noraini Seman, Liyana Ahamad Fouzi, Firdaus Abdul Hamid, Tengku Alina Tengku Ismail

**Affiliations:** 1 Department of Community Medicine, Universiti Sains Malaysia Pusat Pengajian Sains Perubatan, Kubang Kerian, Malaysia; 2 Clinical Research Centre, Hospital Sultanah Nur Zahirah, Ministry of Health, Kuala Terengganu, Malaysia; 3 Department of Non-communicable Disease Epidemiology, London School of Hygiene & Tropical Medicine, London, UK; 4 Department of Communities and Education, Northumbria University, Newcastle Upon Tyne, UK; 5 Department of Neurosciences, Universiti Sains Malaysia—Kampus Kesihatan, Kubang Kerian, Malaysia; 6 Department of Medicine, Hospital Raja Perempuan Zainab II, Ministry of Health, Kota Bharu, Kelatan, Malaysia; 7 Department of Rehabilitation, Hospital Raja Perempuan Zainab II, Ministry of Health, Kota Bharu, Kelatan, Malaysia; 8 Department of Medicine, Hospital Sultanah Nur Zahirah, Ministry of Health, Kuala Terengganu, Malaysia; 9 Clinical Research Centre, Hospital Sultanah Nur Zahirah, Kuala Terengganu, Malaysia

**Keywords:** Stroke, Mobile Applications, Caregivers, Health Education

## Abstract

**Abstract:**

**Background:**

Stroke is one of the top causes of disability in Malaysia, yet caregivers have limited access to structured, culturally tailored education to support poststroke care.

**Objectives:**

To develop and validate the CaknaStrok Education Package (CEP), a blended learning intervention comprising a printed guidebook and a trilingual mobile health application for informal stroke caregivers in Malaysia.

**Design:**

Methodological study involving the development and validation of a caregiver education programme guided by the Analyse, Design, Develop, Implement, Evaluate (ADDIE) instructional design framework.

**Setting:**

Development and validation were conducted in Malaysia between January 2022 and December 2023. Both experts and caregivers were recruited from two tertiary hospitals on the East Coast of Malaysia, with caregivers identified from inpatient wards and outpatient clinics at these hospitals.

**Participants:**

Content validation involved 10 multidisciplinary experts. Face validation involved 14 informal stroke caregivers who met eligibility criteria, and all completed the study.

**Methods:**

CEP was developed based on prior needs assessment and expert input. Content validation was undertaken using the Content Validity Index (CVI) and face validation using the Face Validity Index (FVI), both assessed on a four-point Likert scale. Qualitative feedback was also obtained from the participants.

**Results:**

CEP consists of six modules delivered via a printed guidebook and a trilingual app with videos, assessment tools and local resources. Experts rated the content highly valid (Scale-level (S)-CVI/the average method (Ave): 0.97–0.99 across domains). Caregivers reported strong acceptability (S-FVI/Ave: 0.95–0.99). Qualitative feedback from experts and caregivers informed refinements to content clarity, usability and presentation, including improved navigation, consistent language use and enhanced visual design. Suggestions requiring substantial structural changes were documented for future iterations.

**Conclusions:**

The CEP shows strong content and face validity as a blended caregiver education tool. By combining printed and digital formats, CEP addresses cultural and access challenges and provides a scalable model for stroke caregiver education in Malaysia. Further pilot or feasibility studies are warranted to evaluate usability, engagement and implementation in real-world settings prior to effectiveness evaluation.

**STRENGTHS AND LIMITATIONS OF THIS STUDY**

- The CaknaStrok Education Package was systematically designed and developed using the ADDIE instructional design framework, providing a structured and theory-informed methodological approach.
- Multidisciplinary stroke care experts and informal caregivers were involved in the design and validation processes, enhancing methodological rigour and contextual relevance.
- A blended delivery format (printed guidebook and trilingual mobile health application) was employed to accommodate varying levels of digital access and literacy among caregivers.
- The study focused on design and initial content and face validation; effectiveness, behavioural outcomes and clinical impact were not evaluated at this stage.
- Content and face validation were conducted with a relatively small sample from two tertiary hospitals, which may limit generalisability, and formal readability testing was not undertaken.

## Introduction

 Stroke is one of the most significant public health issues worldwide, and in Malaysia, it ranks among the top five causes of death and disability.^1 2^ Due to the continuously rising number of stroke survivors, the demand for sustained, coordinated poststroke care, particularly within community and home settings, has become increasingly crucial. Yet, the transition from hospital to home often leaves informal caregivers, typically family members, feeling overwhelmed, as they are typically untrained and unprepared to manage the complex care needs of stroke survivors.^3^,^7^ In most cases, the caregiving burden leads to stress, depression and deterioration in the quality of life for both the caregiver and the stroke survivor.^6^,^11^

Despite the increasing attention to stroke rehabilitation services in Malaysia, the structured, evidence-based and culturally tailored educational support for informal caregivers is still lacking.^12^,^15^ In health intervention research, cultural adaptation refers to the systematic process of modifying interventions to ensure alignment with the beliefs, norms, language and everyday practices of a specific target population.^16^ Within digital health interventions, cultural sensitivity encompasses both surface-structure adaptations, such as language, visuals and delivery formats that reflect the target audience and deep-structure adaptations that address the broader cultural, social and contextual factors shaping how health information is understood, accepted and acted on.^17^ Although some general health education materials are available, many remain limited in comprehensiveness, accessibility and contextual relevance, thereby constraining their effectiveness in empowering informal caregivers.^3 18 19^

Several stroke education manuals and mobile applications have been developed by international stroke organisations and research groups.^20^,^28^ However, most are designed for high-income settings, are predominantly English-based and provide limited contextual relevance to Malaysian caregiving practices and healthcare pathways.^23 24^ Locally available materials are often fragmented, discipline-specific or focused primarily on patients rather than caregivers.^23^ This highlights the need for a structured, culturally adapted and caregiver-focused educational package that integrates practical caregiving guidance with locally relevant health system navigation.

This phase focuses on the Design and Development stages, addressing the current gap in culturally relevant and user-friendly educational resources for stroke caregiving. The objective is to develop the CaknaStrok Education Package (CEP), which includes an educational intervention in the form of a printed guidebook and a timetable, as part of the stroke caregiver education module. This guidebook is intended for use alongside a predeveloped mobile health (mHealth) application and was subjected to face and content validation by a panel of multidisciplinary experts.

## Methods

### Study design

This study was a methodological study involving the development and validation of the CEP. The validation phase employed a cross-sectional design, in which content validation was conducted with a panel of multidisciplinary experts and face validation was carried out with informal stroke caregivers at a single point in time. In this study, the term experts refers to multidisciplinary healthcare professionals involved in stroke care, including neurologists, rehabilitation physicians, occupational therapists, physiotherapists and rehabilitation nurses, who were actively involved in stroke patient management for a minimum of 1 year, were currently practising in clinical settings and had received formal professional training in stroke management as part of their clinical practice. Caregivers refer to informal family members providing unpaid care to stroke survivors. The term participants is used only when referring collectively to experts and caregivers.

### Setting

The study was conducted in Malaysia between January 2022 and December 2023. The development of the CEP was carried out during the first phase (January 2022–June 2023) and the second phase, comprising content validation by experts and face validation with caregivers, was conducted between July 2023 and December 2023.

### Package development

This study was part of a multiphase project guided by the Analyse, Design, Develop, Implement, Evaluate (ADDIE) framework instructional design framework, which comprises five phases: analysis, design, development, implementation and evaluation.^29^ The primary objective of this project was to develop an intervention to support informal caregivers in assuming the caregiving role following hospital discharge, with a focus on the early postacute and community care phases after stroke, particularly for stroke survivors with mobility limitations or those who are bed-bound.^30^ The current paper focuses on Phase 2, which involved the design and development of the CEP and its subsequent validation.

In Phase 1, a comprehensive needs assessment was conducted to explore the informational gaps, caregiving challenges and support requirements of stroke caregivers in Malaysia.^3^ A mixed-methods approach was employed, involving in-depth interviews with caregivers and healthcare providers, as well as surveys conducted among healthcare providers. Findings highlighted key issues, including limited caregiver knowledge of stroke management, restricted access to structured educational resources and the need for culturally sensitive, user-friendly and accessible materials. These results, as previously reported, provided the evidence base for the development of the educational package.^3^

Following the findings from Phase 1, Phase 2 aligned with the design and development phases of the ADDIE instructional design framework, emphasising the systematic planning, organisation and creation of the CEP. To accommodate diverse user contexts, the CEP was developed in two complementary formats: (1) a mHealth application offering interactive and accessible content, that was named as CaknaStrok app and (2) a printed guidebook, CaknaStrok: Home Care Guidebook for Stroke Patients serving as a supplementary resource, supporting caregivers in using the app while also offering expanded content beyond the digital version.

During the design phase, the caregiver needs identified in Phase 1 were translated into a structured educational framework tailored to the Malaysian context. The content was designed to be evidence-based, comprehensive, user-friendly and culturally appropriate. Specific learning objectives were established for each module section to guide content development and ensure alignment with caregiver needs. These objectives were refined through input from the expert panel comprised of two neurologists (n=2), rehabilitation physicians (n=2), physiotherapists (n=4), occupational therapists (n=3), nurses (n=2) and representatives from stroke support organisations (n=3). Informal caregivers (n=3) were also engaged in the review process to enhance the practicality, relevance and cultural sensitivity of the materials.

During the development phase, content mapping was undertaken to align identified caregiver needs with established stroke care guidelines and expert recommendations. Draft materials underwent iterative review by key stakeholders to assess accuracy, completeness and appropriateness. The development process included three in-person meetings conducted in a workshop format over two consecutive days, during which structured content review, group discussion and iterative refinement of module content, delivery formats and supporting materials were carried out. This process enabled real-time expert feedback and informed successive revisions of the educational package. Preliminary versions of the mHealth application and guidebook were then produced and refined through pilot drafting. These prototypes underwent structured content validation and face validation to ensure quality, clarity and usability before finalisation.

After this phase, the next steps in the ADDIE model will include implementation, whereby the package will be piloted in healthcare settings, and evaluation, aimed at assessing usability as well as its effects on caregiving burden, emotional health and quality of life.

### Module validation

As mentioned above, to ensure the CEP was relevant, accurate and appropriate for its target audience, both content and face validity assessments were conducted. The mobile application and printed guidebook were evaluated together as a single integrated package to reflect their intended combined use.

### Content validation

The content validity of the CEP was conducted by using a purposive sampling strategy. 10 experts were recruited, following established guidelines suggesting that a panel size of 6–10 experts is sufficient to achieve stable Content Validity Index (CVI) estimates.^31^ To ensure the credibility of the validation process and minimise potential bias, a panel of experts was recruited from two institutions on the east coast of Malaysia, by identifying them through consultation with the heads of their respective departments. Subsequently, they were contacted to brief on the study objectives and procedures. An experienced informal caregiver was also included to contribute practical perspectives, who served as the primary caregiver for a stroke survivor for 1 year and possessed adequate language proficiency to participate in the validation process. On consent to participate, appointments were arranged for the study procedure. On the appointment day, written informed consent was obtained first. Then, they were requested to install the CaknaStrok app on their device and received brief training on its features and functions. They were also provided with the printed CaknaStrok: Home Care Guidebook for Stroke Patients. Participants were given up to 3 days to review the package and rate its relevance, clarity and comprehensiveness. Each item was scored using a four-point Likert scale (1=not relevant, 2=somewhat relevant, 3=relevant and 4=very relevant). This review period was selected pragmatically to accommodate experts’ availability and reflects common practice in content validation studies, which are designed to assess item relevance and clarity through expert review rather than long-term engagement.^31^ To minimise participant burden and reduce the risk of attrition, the review period was kept brief; however, participants who required additional time were allowed to extend their review prior to submitting their ratings.

The validation process involved calculating the Item-level Content Validity Index (I-CVI) and the Scale-level Content Validity Index (S-CVI) based on methods outlined by Yusoff.^32^ The I-CVI was derived as the proportion of experts assigning a rating of 3 or 4 to each item. For the S-CVI, two metrics were employed: (1) the average method (S-CVI/Ave), representing the mean I-CVI across all items, and (2) the universal agreement method (S-CVI/UA), indicating the proportion of items for which all experts gave a rating of 3 or 4. A score of 1.0 reflects perfect agreement among experts, indicating that all rated the item as either ‘quite relevant’ or ‘highly relevant’, thereby representing the highest possible level of content validity. However, a CVI threshold of more than 0.78 was considered acceptable, notably when the number of experts exceeded nine.^32^

### Face validation

For face validation, a convenience sampling method was applied to recruit 14 informal caregivers of stroke patients from Hospital Sultanah Nur Zahirah and Hospital Raja Perempuan Zainab II. This sample size aligns with established recommendations for face validation studies.^33^ Caregivers who accompanied stroke patients in the ward or during routine outpatient follow-up visits were approached and provided with verbal and written explanations of the study. Primary caregivers who expressed interest were given the option to discuss participation with their family members before providing written consent.

On consenting, caregivers were assisted in installing the CaknaStrok app and received instructions on its use. They were also asked to review the printed CaknaStrok: Home Care Guidebook for Stroke Patients. They were given up to 3 days to evaluate the package in terms of understandability. Each item was rated on a four-point Likert scale: 1=not clear or understandable, 2=somewhat clear or understandable, 3=clear and understandable and 4=very clear and understandable. The timeframe of 3 days was selected to accommodate caregivers’ availability and minimise burden, with flexibility for participants who required additional time before completing the face validation assessment. The concise, guidance-oriented design of the package, incorporating short videos and infographic-based content, was intended to support rapid understanding and practical reference rather than prolonged instructional engagement.^34^

Face validity was assessed using the Item-level Face Validity Index (I-FVI) and the Scale-level Face Validity Index (S-FVI) as described by Yusoff. The I-FVI was calculated by dividing the number of respondents rating an item as 3 or 4 by the total number of raters. Similarly, the S-FVI was assessed using two approaches: the S-FVI/Ave method, which calculates the mean of all I-FVI scores, and the S-FVI/UA method, representing the proportion of items that received unanimous ratings of 3 or 4. A score of 1.0 represents perfect agreement among raters, indicating that all participants rated the item as either ‘clear’ or ‘very clear’, and thus reflects the highest possible level of face validity. However, a minimum score of 0.80 was set as the acceptable threshold for both I-FVI and S-FVI metrics.^33 34^

 In addition to the quantitative ratings, participants were invited to provide open-ended comments on the clarity, relevance and usability of the CEP. These open-ended comments were analysed descriptively by grouping similar responses and identifying recurring issues related to clarity, usability and content relevance. This approach was employed to directly inform practical refinement of the package rather than to generate formal, theoretical qualitative themes.

### Patient and public involvement

Patients were not involved in setting the research question, study design or outcome measures. However, informal stroke caregivers contributed during the validation stage by providing feedback to refine the educational package.

## Results

### Module development

**Figure 1:**
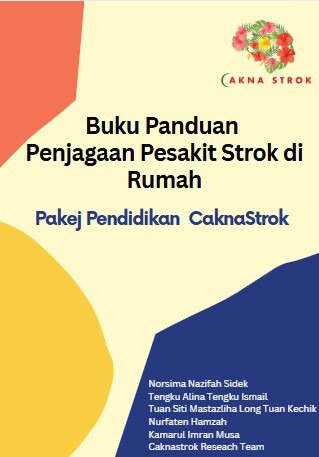
Front page of CaknaStrok guidebook.

**Figure 2:**
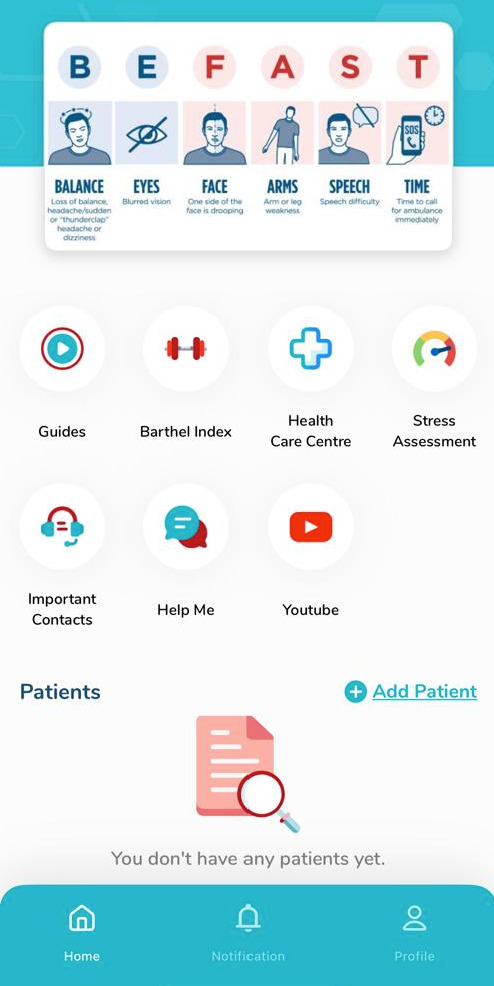
CaknaStrok mobile app homepage.

A primary design objective of the CEP was to maximise accessibility for caregivers with varying levels of literacy. The CEP was developed as a blended educational resource comprising printed materials (guidebook) (figure 1) and digital content delivered via the CaknaStrok mobile application (figure 2). To address language demands, all written and audiovisual content was developed using plain, non-technical language, short sentences and commonly used terminology within Malaysian healthcare settings. Complex medical terms were avoided where possible or explained using simple descriptions, and key messages were reinforced through infographic-based layouts and short video segments to reduce reliance on dense text and support rapid comprehension.

Linguistic accessibility was further addressed through the use of multiple language formats. The printed guidebook was produced in Malay, as Malay (Bahasa Malaysia/Bahasa Melayu) is the national and official language of Malaysia and is taught as a compulsory subject and used as the primary medium of instruction within the national education system,^35^ thereby ensuring broad comprehension across caregiver populations. The mobile application was developed in three languages: Malay, English and Mandarin to enhance reach among Malaysia’s linguistically diverse caregivers. Although Tamil is widely spoken within the Indian community, this group represents a smaller proportion of the Malaysian population compared with Malay- and Mandarin-speaking communities; consequently, Tamil localisation was not implemented during the initial development phase but is planned for future iterations to further enhance linguistic inclusivity.^36^

Formal readability testing using established readability indices was not undertaken at this stage. Instead, readability was addressed pragmatically during development through iterative expert review, with particular attention to wording simplicity, clarity of instructions and visual presentation. Translation was managed using a forward-translation approach by bilingual members of the research team with clinical backgrounds, followed by review by healthcare professionals fluent in the target languages to ensure conceptual equivalence rather than literal translation.

The guidebook was structured into six main content modules, each addressing specific domains relevant to stroke caregiving. Before the module content, the guidebook includes introductory sections comprising the *Latar Belakang Pembangunan Aplikasi CaknaStrok* (Background of CaknaStrok App Development), *Pengenalan* (Introduction), the objectives of the module development, the target audience and *Panduan Penggunaan Modul* (Guide for Module Use). The details of each module are presented in table 1. In addition to the six modules, an exercise timetable was included as a practical tool to promote routine physical rehabilitation activities. The timetable was developed with input from rehabilitation therapists and is tailored to home-based implementation, allowing caregivers to assist stroke survivors.

**Table 1:** Topics, objectives, contents and delivery methods of the CaknaStrok guidebook

Modules	Topic	Objectives	Content	Delivery method
1	Stroke in general	Provide a basic understanding of what a stroke is, the types of strokes and how it occurs. Increase awareness of risk factors and early signs of stroke. Foster understanding of the importance of early treatment in improving recovery outcomes.	What is a stroke Expectations of stroke patients in the hospital Impact of stroke and patient needs Guide on stroke patients’ feeding tubes Type of stroke Stroke symptom Stroke risk factors Dietary guide	Video (in app) Video (in app) Video (in app) Video (in app) Guidebook Guidebook Guidebook Guidebook
2	Stroke complications and prevention	Explain the complications that stroke patients, such as physical disabilities, cognitive problems and communication difficulties, may face. Guide preventive measures to reduce the risk of recurrent stroke. Empower caregivers and patients with knowledge on managing stroke-related complications.	Stroke complications and prevention Bed sores and preventive measures Preventing recurrent stroke	Guidebook and video (in App) Video (in app) Guidebook and video (in app)
3	Role of the therapist	Introduce various types of therapy that are important in stroke rehabilitation, including physiotherapy and occupational therapy. Emphasise the importance of a continuous and personalised rehabilitation programme tailored to the individual patient’s needs. Enhance awareness of how therapy can aid in the recovery of physical and cognitive functions in stroke patients.	Importance of poststroke therapy Stroke care and targeted therapy List of the nearest healthcare facilities National Stroke Association of Malaysia (NASAM) contact number Index Barthel	Video (in app) and guidebook Video (in app) App App Guidebook and app
4	Role of caregiver	Outline the primary responsibilities of caregivers in managing stroke patients at home. Provide practical guidance on how caregivers can support the patient’s recovery process. Develop caregivers’ skills in managing emotions and stress, as well as self-care.	Understanding personal needs as a caregiver Tips for stroke caregivers	Video (in app) Video (in app)
5	Stroke and mental health	To provide information on the impact of stroke on the mental health of patients and caregivers. To offer guidance on managing mental health issues such as depression, anxiety and poststroke stress. To emphasise the importance of psychosocial support in the overall recovery of stroke patients.	Stroke from the mental health aspect Patient emotions Dealing with emotional burden Internal breathing technique Stress assessment	Video (in app) Video (in app) Video (in app) Video (in app) Guidebook and app
6	Support for stroke survivors and caregivers	Provide information about available support resources for stroke patients and caregivers, including healthcare services, financial aid and support communities. Raise awareness of the rights and assistance accessible to patients and caregivers. Encourage caregivers and patients to seek and use available support to assist in stroke care.	Stroke patients’ follow-up care Domiciliary healthcare service Stroke patient aid Introduction to a non-governmental organisation for stroke patients	Video (in app) Video (in app) Video (in app) Video (in app)

The app was structured by module and contained 19 educational videos (ranging from 2 to 7 min in duration) covering key areas: poststroke education, general caregiving techniques and support resources for caregivers (including emotional, domiciliary and non-governmental organisation (NGO) support). Additionally, it featured 13 short therapy tutorials (lasting 1 to 2 min), which served as practical video guides that caregivers could easily refer to for specific techniques and instructions. In addition to the video, the app also includes the Barthel Index assessment tool, a list of the nearest healthcare centres, stress assessment tools and an important contact for the caregiver.

This educational package was designed for flexible, self-paced use. Caregivers could access modules non-sequentially, pause and replay videos, and prioritise content relevant to their immediate caregiving needs. The current version prioritises physical rehabilitation as the initial focus to facilitate functional recovery. The psychological and mental health components were not incorporated in this phase to maintain a focused scope and ensure feasibility. Nonetheless, these components will be considered in future expansions of the programme to provide a more holistic approach to stroke rehabilitation.

Cultural relevance of the CEP was evaluated through alignment of module content with caregiver needs identified in the prior needs assessment and through structured feedback from multidisciplinary experts and informal caregivers.^3 37^ As presented in table 1, module topics and objectives were mapped to caregivers’ expressed priorities, including stroke education, practical caregiving skills, mental and emotional support, and access to community and non-governmental resources. Contextual relevance was further assessed through the use of locally familiar language, Malaysian healthcare pathways and nationally available services embedded within the content.

### Content validation

The CEP was reviewed and evaluated by 10 expert panel members. Overall, content validation demonstrated a high level of agreement across all domains. For the video component, the S-CVI/Ave was 0.99 for relevancy, 0.98 for clarity and 0.96 for completeness, with all items rated as acceptable. The app content achieved S-CVI/Ave scores of 0.95 for relevancy, 0.99 for clarity and 0.97 for completeness, also indicating strong content validity. Similarly, the guidebook obtained S-CVI/Ave scores of 1.0 for relevancy, 0.98 for clarity and 0.98 for completeness. A detailed summary of the CaknaStroke mobile app and guidebook results, respectively, is presented in tables2  3. Collectively, these findings confirm that the developed educational materials are relevant, explicit and sufficiently comprehensive to support caregivers of individuals with stroke.

**Table 2:** CVI of the CaknaStrok Education Package (CaknaStrok mobile app)

Domain 1 (video)	Item	Relevancy (I-CVI)	Clarity (I-CVI)	Completeness (I-CVI)	Interpretation
**General tips**					
Video 1	What is a stroke	1	1	1	Item acceptable
Video 2	Expectations of stroke patients in the hospital	1	1	1	Item acceptable
Video 3	Impact of stroke and patient needs	1	1	0.9	Item acceptable
Video 4	Guide on stroke patients’ feeding tubes	1	1	0.8	Item acceptable
Video 5	Stroke complications and prevention	1	1	1	Item acceptable
Video 6	Bed sores and preventive measures	1	0.9	0.8	Item acceptable
Video 7	Preventing recurrent stroke	1	1	0.9	Item acceptable
Video 8	Importance of poststroke therapy	1	1	1	Item acceptable
Video 9	Stroke care and targeted therapy	1	1	1	Item acceptable
Video 10	Understanding personal needs as a caregiver	1	1	1	Item acceptable
Video 11	Tips for stroke caregivers	1	1	1	Item acceptable
Video 12	Stroke from the mental health aspect	1	1	1	Item acceptable
Video 13	Patient emotions	0.9	0.9	0.9	Item acceptable
Video 14	Dealing with emotional burden	1	1	1	Item acceptable
Video 15	Internal breathing techniques	1	1	1	Item acceptable
Video 16	Stroke patient follow-up care	1	0.9	0.8	Item acceptable
Video 17	Domiciliary Healthcare Services	1	1	1	Item acceptable
Video 18	Kelantan State Stroke Association	1	1	1	Item acceptable
Video 19	Stroke patient aid	1	1	1	Item acceptable
**Tutorial**					
**Fully dependent**					
Video 1	Bed positioning	1	1	1	Item acceptable
Video 2	Bed mobility	0.9	0.8	0.8	Item acceptable
Video 3	From bed to wheelchair	1	1	0.9	Item acceptable
Video 4	Nasogastric tube feeding	1	1	1	Item acceptable
Video 5	Bed bath	1	1	1	Item acceptable
Video 6	Putting on clothes for bedridden patients	1	1	1	Item acceptable
Video 7	Putting on a diaper	1	1	1	Item acceptable
Video 8	Changing diaper	1	1	1	Item acceptable
**Semidependant**					
Video 9	Putting on a shirt	1	1	1	Item acceptable
Video 10	Taking off a shirt	1	1	1	Item acceptable
Video 11	Putting on a T-shirt	1	1	1	Item acceptable
Video 11	Taking off a T-shirt	1	1	1	Item acceptable
Video 12	Putting on pants	1	1	1	Item acceptable
Video 13	Self-feeding	1	1	1	Item acceptable
Video S-CVI/Ave		0.99	0.98	0.96	Video acceptable

Domain 2 (app content)	Item	Relevancy (I-CVI)	Clarity (I-CVI)	Completeness (I-CVI)	Interpretation
1	Registration	0.9	1	0.9	Item acceptable
2	Profile	1	1	1	Item acceptable
3	Notification	1	1	1	Item acceptable
4	Guides	0.9	1	1	Item acceptable
5	Barthel Index	0.9	1	0.8	Item acceptable
6	Health Care Centres	1	1	1	Item acceptable
7	Stress Assessment	1	1	1	Item acceptable
8	Important Contacts	0.9	1	0.9	Item acceptable
9	Help Me	1	1	1	Item acceptable
10	Youtube	0.9	0.9	1	Item acceptable
11	Patients	0.9	1	1	Item acceptable
12	Dashboard	1	1	1	Item acceptable
App content S-CVI/Ave		0.95	0.99	0.97	App content acceptable

I-CVI, Item-level Content Validity Index; S-CVI/Ave, Scale-level Content Validity Index/average method.

**Table 3:** CVI of the CaknaStrok Education Package (CaknaStrok guidebook)

Domain 3 (guidebook)	Item	Relevancy (I-CVI)	Clarity (I-CVI)	Completeness (I-CVI)	Interpretation
Module 1	Stroke in general	1.0	1.0	1.0	Item acceptable
Module 2	Stroke complications and prevention	1.0	1.0	1.0	Item acceptable
Module 3	Role of the therapist	1.0	1.0	1.0	Item acceptable
Module 4	Role of caregiver	1.0	1.0	1.0	Item acceptable
Module 5	Stroke and mental health	1.0	1.0	1.0	Item acceptable
Module 6	Support for stroke survivors and caregivers	1.0	1.0	1.0	Item acceptable
Appendix 1	Home exercise timetable	1.0	0.8	0.8	Item acceptable
Appendix 2	Frequently asked questions	1.0	1.0	1.0	Item acceptable
Guidebook S-CVI/Ave		1.0	0.98	0.98	Guidebook acceptable

I-CVI, Item-level Content Validity Index; S-CVI/Ave, Scale-level Content Validity Index/average method.

Based on qualitative feedback, several areas for refinement were identified within the CEP from an initial draft to a final version. Revisions implemented in response to this feedback addressed both content and usability issues. For the guidebook, reviewers highlighted the need for clearer structure and instructions, particularly for the home exercise schedule. In response, titles were clarified, step-by-step guidance was added, and examples of completed forms were provided to support practical use. Given that many caregivers were older adults, font sizes were enlarged, and medical terminology was simplified to improve accessibility, including replacing less familiar terms (eg, ‘health facilities’) with commonly used references such as ‘hospital’ or ‘health clinic’. Infographic-based layouts were also enhanced to reduce reliance on text-heavy explanations.

For the mHealth application, qualitative feedback identified technical and usability issues, including registration difficulties, video playback interruptions and unclear navigation pathways. Usability refinements included streamlining navigation, correcting non-functional back buttons, standardising language use across interfaces, updating contact links and reorganising video content to improve logical flow. The ‘Help Me’ feature was perceived as unclear; consequently, it was refined to focus exclusively on emergency use (999), supported by confirmation prompts, while healthcare facility listings and resource links were clarified to better guide users seeking professional support. A dedicated project contact email was also added to improve communication pathways.

Some feedback could not be fully incorporated within the current phase due to feasibility and technical constraints. These included requests for expanded nationwide facility listings, additional professional representation in video content, further language customisation and major structural changes requiring application version updates. Such feedback was documented and will inform future iterations of the package following pilot and feasibility testing.

### Face validation

The face validation process involved 14 target users, all of whom were informal caregivers. All 14 caregivers interacted with both components of the educational package, including the mobile application and the printed guidebook, prior to completing the face validation assessment. Across the video, app content and guidebook components, all items demonstrated high ratings for comprehensibility and clarity, with I-CVI values ranging from 0.93 to 1.00. The video component achieved an overall S-FVI/Ave of 0.99, the app content 0.95 and the guidebook 0.99. This finding supports the retention of all items without modification as detailed in table 4 for the mobile app and table 5 for the guidebook.

**Table 4:** FVI of CaknaStrok Education Package (mobile app)

Domain 1 (video)	Item	Understandability and clarity (I-CVI)	Interpretation
**General tips**			
Video 1	What is a stroke	0.93	Item acceptable
Video 2	Expectations of stroke patients in the hospital	1.00	Item acceptable
Video 3	Impact of stroke and patient needs	1.00	Item acceptable
Video 4	Guide on stroke patients’ feeding tubes	0.93	Item acceptable
Video 5	Stroke complications and prevention	0.93	Item acceptable
Video 6	Bed sores and preventive measures	1.00	Item acceptable
Video 7	Preventing recurrent stroke	1.00	Item acceptable
Video 8	Importance of poststroke therapy	1.00	Item acceptable
Video 9	Stroke care and targeted therapy	1.00	Item acceptable
Video 10	Understanding personal needs as a caregiver	1.00	Item acceptable
Video 11	Tips for stroke caregivers	1.00	Item acceptable
Video 12	Stroke from mental health aspect	1.00	Item acceptable
Video 13	Patient emotions	1.00	Item acceptable
Video 14	Dealing with emotional burden	0.93	Item acceptable
Video 15	Internal breathing techniques	1.00	Item acceptable
Video 16	Stroke patient follow-up care	1.00	Item acceptable
Video 17	Domiciliary healthcare services	1.00	Item acceptable
Video 18	Kelantan State Stroke Association	1.00	Item acceptable
Video 19	Stroke patient aid	1.00	Item acceptable
**Tutorial**			
**Fully dependent**			
Video 1	Bed positioning	1.00	Item acceptable
Video 2	Bed mobility	1.00	Item acceptable
Video 3	From bed to wheelchair	1.00	Item acceptable
Video 4	Nasogastric tube feeding	1.00	Item acceptable
Video 5	Bed bath	1.00	Item acceptable
Video 6	Putting on clothes for bedridden patients	1.00	Item acceptable
Video 7	Putting on a diaper	1.00	Item acceptable
Video 8	Changing diaper	1.00	Item acceptable
**Semidependant**			
Video 9	Putting on a shirt	1.00	Item acceptable
Video 10	Taking off a shirt	1.00	Item acceptable
Video 11	Putting on a T-shirt	1.00	Item acceptable
Video 11	Taking off a T-shirt	1.00	Item acceptable
Video 12	Putting on pants	0.93	Item acceptable
Video 13	Self-feeding	1.00	Item acceptable
Video S-FVI/Ave		0.99	Video acceptable

**Domain 2** **(APP content)**	**Item**	**Understandability and clarity** **(I-CVI)**	**Interpretation**
1	Registration	0.93	Item acceptable
2	Profile	1.00	Item acceptable
3	Notification	1.00	Item acceptable
4	Guides	0.93	Item acceptable
5	Barthel Index	0.93	Item acceptable
6	Health Care Centres	1.00	Item acceptable
7	Stress Assessment	1.00	Item acceptable
8	Important Contacts	1.00	Item acceptable
9	Help Me	1.00	Item acceptable
10	Youtube	1.00	Item acceptable
11	Patients	1.00	Item acceptable
12	Dashboard	1.00	Item acceptable
App content S-FVI/Ave		0.95	App content acceptable

I-CVI, Item-level Content Validity Index; S-FVI/Ave, Scale-level Face Validity Index/average method.

**Table 5:** FVI of CaknaStrok Education Package (guidebook)

Domain 3 (guidebook)	Item	Understandability and clarity (I-CVI)	Interpretation
Module 1	Stroke in general	1.00	Item acceptable
Module 2	Stroke complications and prevention	1.00	Item acceptable
Module 3	Role of the therapist	1.00	Item acceptable
Module 4	Role of caregiver	1.00	Item acceptable
Module 5	Stroke and mental health	1.00	Item acceptable
Module 6	Support for stroke survivors and caregivers	0.93	Item acceptable
Appendix 1	Home exercise timetable	1.00	Item acceptable
Appendix 2	Frequently asked questions	1.00	Item acceptable
Guidebook S-FVI/Ave		0.99	Guidebook acceptable

I-CVI, Item-level Content Validity Index; S-FVI/Ave, Scale-level Face Validity Index/average method.

Qualitative feedback from caregivers was predominantly positive, with no negative comments reported regarding the relevance or usefulness of the educational content. Caregivers highlighted the interactive design of both the mobile application and the printed guidebook, describing the content as explicit, practical and helpful in enhancing their understanding of stroke care. Several participants noted that the structured layout of the guidebook facilitated easier navigation of the accompanying mHealth application, supporting its use as a practical reference during caregiving.

Minor usability issues were, however, identified during the qualitative review, primarily related to application navigation. These included instances where back buttons within the ‘Important Contacts’ and ‘YouTube’ sections did not redirect users appropriately within the app. These issues were technical in nature and unrelated to content quality and were subsequently communicated to the technical development team for resolution as part of usability refinements.

Content and face validation findings demonstrated high ratings for clarity, relevance and acceptability across modules, indicating that the educational materials are clear, comprehensible and appropriate for the intended caregiver audience, while also supporting their cultural acceptability within the Malaysian caregiving context.

## Discussion

The CEP represents the first locally developed stroke education package in Malaysia that integrates both printed and digital components specifically tailored for informal stroke caregivers. This study outlines the systematic design and development of the CEP, which comprises a comprehensive printed guidebook and a predeveloped, trilingual mHealth application. The need for such a resource is underscored by a persistent gap in the Malaysian context. A recent narrative review indicates that existing stroke-related digital interventions have largely focused on acute, hospital-based stroke management (eg, Regional Emergency Stroke Quick-Response Network) or early detection (eg, Stroke Pre-Detection mobile application), rather than postdischarge or home-based caregiving.^24^ The absence of a structured educational resource specifically targeting informal stroke caregivers highlights this critical need and supports the development of a caregiver-centred package like the CEP.

The design of the CEP was fundamentally informed by an earlier needs assessment^3 37^ and published literature,^6 14 23 24^ which consistently highlighted substantial knowledge gaps among stroke caregivers. These gaps included a lack of structured, culturally adapted and locally relevant educational materials^3 23 24 38^. The overall development of the CEP was rigorously guided by the ADDIE instructional design model,^29^ and this paper specifically focuses on the Design and Development phases. To address the identified gaps, this process emphasised contextual relevance and accessibility. This was achieved by incorporating essential input from multidisciplinary healthcare experts and caregivers to ensure the content’s accuracy, cultural appropriateness and practicality in everyday caregiving.

Consequently, the CEP was developed with a focus on explicit, meaningful and step-by-step content culturally tailored for caregivers through multilingual delivery, simplified language, culturally familiar caregiving practices and alignment with Malaysian healthcare structures. The printed guidebook, for example, was structured into thematic sections covering stroke education, daily care tasks, emotional and psychosocial support, rehabilitation roles and strategies, and navigation of the healthcare system. Each section was accompanied by specific learning objectives, corroborating evidence that cultural tailoring and instructional structuring improve the comprehension and usability of health education materials.

A notable strength of CEP lies in its blended learning format, which integrates printed resources with a trilingual app. While previous interventions often relied exclusively on either print or digital materials, CEP bridges the digital divide by ensuring accessibility for caregivers in rural or underserved areas with limited smartphone access, while also providing interactive features such as video demonstrations, assessment tools and local healthcare directories for those with higher digital literacy. Infographics and step-by-step instructions in the guidebook were specifically incorporated to overcome literacy challenges, consistent with recommendations from published literature on effective health communication design.^39^,^41^ This dual-format design reflects growing evidence that multicomponent interventions, which combine educational materials with interactive and supportive components, can significantly enhance caregiver engagement, competency and well-being compared with single-format approaches.^42 43^

The development phase of the CEP integrated both content and face validation to ensure clinical accuracy, relevance and ease of use for caregivers. The incorporation of these validation methods was essential to ensure the reliability and practicality of the educational module. Similar validation approaches have been widely adopted in the development of other health education materials to enhance their quality and usability.^44^,^46^

The validation process confirmed the robustness of CEP. Content validation demonstrated strong expert agreement, with the S-CVI/Ave surpassing the widely accepted threshold of 0.78.^47^ The guidebook achieved perfect scores for relevance and near-perfect scores for clarity and completeness, surpassing those reported for a Rabies Health Education Module,^46^ Universiti Sains Malaysia-Insulin Adherence Module for Patients with Type 2 Diabetes Mellitus,^44^ Breastfeeding and Dietary Education Package for gestational diabetes mellitus women^48^ and comparable to results achieved in educational interventions for an intimate partner violence module for young adults, IPV educational module,^45^ as well as for aboriginal primary school students, ‘Eat Right, Future Bright: Nutrition Education Programme’ module.^49^

The qualitative feedback on the mHealth application revealed important areas for refinement. Technical difficulties, such as registration errors and video playback interruptions, were primarily attributed to internet connectivity issues, a notably challenging problem frequently reported in mHealth interventions.^23 50 51^ These issues nonetheless highlight the need for offline functionalities or lightweight formats to enhance usability in resource-limited contexts. Within this study, distinct support pathways were established for technical and professional assistance: technical issues related to application functionality were managed by the research team responsible for application maintenance and were accessible via the in-app contact interface, whereas caregiving and clinical queries were supported through embedded healthcare and community resources, including Ministry of Health facilities, rehabilitation services and relevant NGOs.

However, the ‘Help Me’ feature was perceived as unclear, indicating the need for clearer differentiation and labelling of these support pathways, including more explicit representation of available professional resources and healthcare facilities. Suggestions also included more transparent labelling of healthcare facilities and an expanded contact directory, underscoring the crucial role of navigation support in enhancing the usability and effectiveness of caregiver education tools. This finding is consistent with previous research, which highlights that responsive communication features are essential for building caregiver confidence and fostering trust in digital health tools. Additionally, well-structured directories and clearly presented healthcare pathways further enhance user engagement.^52 53^

Similarly, expert feedback on the guidebook pointed to the need for greater clarity in the ‘Home Exercise Timetable’, particularly through the inclusion of practical examples and more explicit instructions. Additional suggestions included using larger fonts for improved readability and simplifying terminology by employing commonly understood local healthcare terms. These refinements were incorporated before face validation to strengthen the clarity, usability and cultural appropriateness of the guidebook.

Face validation findings reinforced the clarity and relevance of CEP. The S-FVI/Ave exceeded the recommended 0.90 suggested by Patel and Desai,^33^ confirming its suitability for end users. Caregivers reported that the guidebook’s interactive layout and simplified language enhanced their understanding of stroke care and facilitated use of the accompanying mobile application. Their feedback demonstrated that the blended design created a complementary and reinforcing effect between the printed and digital components. This integration of analogue and digital resources represents a novel contribution to stroke caregiver education in Malaysia, where interventions often remain fragmented or inaccessible.

The high validity ratings for both content and face validation may be partly attributable to the blended design of the CEP, which integrates a mHealth application with a printed guidebook, as well as the use of infographic-based content and short instructional videos to reduce cognitive load and reliance on dense text. Prior studies have indicated that blended interventions, as well as the use of pictograms and video-based materials, are among the most effective visual strategies in health education interventions, particularly for populations with limited literacy.^20 22 39^ Taken together, these findings indicate that the CEP is a rigorously developed, validated and contextually relevant educational intervention that was effectively tailored to the needs of informal stroke caregivers within the Malaysian context. Rather than individual-level tailoring, the development approach focused on cultural tailoring, primarily at the surface-structure level, informed by caregivers’ priorities identified through the prior needs assessment and refined through iterative engagement with multidisciplinary experts and caregivers. The selection and organisation of modules reflect commonly reported caregiver needs, including foundational stroke knowledge, practical caregiving guidance, emotional and mental health support, and access to community and non-governmental resources. Although poststroke depression is a major complication influencing recovery,^54^ it is addressed within the mental health module in the current version to maintain feasibility during the initial development phase; future iterations will integrate poststroke depression more explicitly within the ‘Stroke Complications and Prevention’ module. By incorporating insights from both experts and caregivers, the CEP ensures not only content accuracy but also accessibility and cultural relevance. Beyond language translation, contextual relevance was strengthened through the use of locally familiar terminology, Malaysian healthcare pathways and nationally available support services, consistent with the family-centred nature of stroke care in Malaysia. While validation findings support the overall appropriateness and acceptability of the materials, deeper cultural adaptation addressing underlying belief systems and explanatory models of illness was beyond the scope of the current phase and will be explored in future iterations of the programme.

Through this approach, it helps bridge longstanding gaps in stroke caregiver education and offers a practical, scalable blended solution. While developed specifically for the Malaysian context, the ADDIE-guided development and validation process illustrated in this study may inform future caregiver education initiatives, provided that local healthcare systems, digital access and literacy considerations are carefully addressed.

The validated CEP has important implications for practice and health systems. For clinical practice, it provides structured, culturally relevant guidance that can support caregivers in performing daily care, rehabilitation and emotional support roles more confidently. From a policy perspective, CEP could be integrated into national stroke programmes and linked to the National Stroke Registry, complementing hospital and community-based rehabilitation services. Embedding CEP into primary care and community rehabilitation workflows could enhance continuity of stroke care and reduce caregiver burden. As for research, this study establishes a validated foundation for future evaluations of CEP’s effectiveness in improving caregiver knowledge, confidence and patient outcomes.

This study provides evidence for the systematic development and initial validation of the CEP, demonstrating its clarity, acceptability and contextual relevance for informal stroke caregivers during the postdischarge period in Malaysia. As this work focused on design, development and validation, the findings do not yet establish effectiveness or implementation outcomes. Accordingly, the next step should involve a pilot or feasibility study to assess usability, engagement and practical implementation in real-world settings. These subsequent phases will enable refinement of the intervention and inform the design of future effectiveness evaluations.

Several methodological limitations should be acknowledged. First, by focusing on the design and initial validation of the CEP, this study did not evaluate its effectiveness or clinical impact. Therefore, conclusions regarding behavioural change, caregiver outcomes or patient-related benefits cannot be drawn at this stage. Second, the content and face validation were conducted using a relatively small panel of multidisciplinary experts and a limited number of informal caregivers from two tertiary hospitals. Although the sample sizes are consistent with established recommendations for developmental validity studies, this may restrict the immediate generalisability of the findings. Third, while cultural relevance and accessibility were addressed through multilingual delivery and simplified language, formal readability testing and in-depth deep-structure cultural adaptation were not undertaken. Finally, participants were given a short review period during validation, which may have limited prolonged engagement with the materials. However, this pragmatic approach was adopted to minimise participant burden and attrition, aligning with common practice in validation studies focusing on clarity and relevance rather than long-term use. These acknowledged limitations collectively highlight the necessity for subsequent research to move beyond initial validation and comprehensively evaluate the CEP’s efficacy, implementation fidelity and definitive clinical impact in real-world use.

## Conclusion

This is the first developed and validated CEP that combines a printed guidebook with a mobile application, guided by the ADDIE model. High content and face validity scores confirm its clarity, cultural relevance and practical value for informal caregivers of stroke patients. By bridging print and digital formats, CEP enhances caregiver confidence in both stroke care and app navigation, offering a scalable model for caregiver education and highlighting the potential of blended learning in health education within the Malaysian healthcare system.

## Data Availability

Data are available upon reasonable request.
