# Supplementary material for: Bridging the stroke care gap: development and validation of CaknaStrok Education Package (CEP) for caregivers of stroke survivors in Malaysia
Source: BMJ Open. 2026 Feb 26;16(2):e111268. doi: 10.1136/bmjopen-2025-111268 (PMC12958912; doi:10.1136/bmjopen-2025-111268)
Supplement: Supplementary file 1 [file bmjopen-2025-111268.reviewer_comments.pdf]

PEER REVIEW HISTORY

BMJ Open publishes all reviews undertaken for accepted manuscripts. Reviewers are asked to complete a checklist review form and are provided with free text boxes to elaborate on their assessment. These free text comments are reproduced below.

ARTICLE DETAILS

Title (Provisional)

Bridging The Stroke Care Gap: Development And Validation Of A CaknaStrok Education Package (CEP) For Stroke Caregivers

Authors

Sidek, Norsima Nazifah; Kamalakannan, Sureshkumar; Musa, Kamarul; Long Tuan Kechik, Tuan Siti Mastazliha; Hamzah, Nurfaten; Abd Hamid, Rose Izura; Darus, Daryani; Ibrahim, Khairul Azmi; Seman, Noraini; Ahamad Fouzi, Liyana; Abdul Hamid, Firdaus; Ismail, Tengku Alina Tengku

---

VERSION 1 - REVIEW

**Reviewer**	**1**
**Name**	**Adcock, Amelia**
**Affiliation**	**West Virginia University, Neurology**
**Date**	**04-Nov-2025**
**COI**	**None**

---

this was an excellently designed and well-written presentation of the authors' findings . Appropriate methodology and a meticulous approach were utilized. The findings were clear and represent important work regarding patient level outreach and post-acute care. Without reservation, I would recommend this work be published.

A potential pitfall (acknowledged by the authors) is the small face validation including only 14 informal caregivers. It is furthermore unclear how many of the 14 participants interacted with the app and provided feedback for the package (thus completing the "face validation"). The authors furthermore called out their plan to include a more comprehensive approach in future versions that would include psychological factors. Post-stroke depression is a common complication that prolongs physical recovery from stroke symptoms. Although the authors acknowledge their plan to correct this deficiency, I would suggest that they add post-stroke depression in the "stroke complications and prevention" module as opposed to only discussing it as an isolated "stroke and mental health" topic.

Finally, in regard to the difficulties identified with tech and professional help, it may be helpful to explicitly state what are the resources available for each area (who and what responds to technological queries and who is responding from the health professional side?

**Reviewer**	**2**
**Name**	**Jammal, Melissa**
**Affiliation**	**Western Sydney University, School of Health Sciences**
**Date**	**24-Nov-2025**
**COI**	**None**

Well done on developing a program for carers of people who have experienced a stroke. This is timely, and necessary work and will be an important contribution to the literature. To help further strengthen the manuscript, I've outlined some suggestions below.

General Comments

• The manuscript would benefit from greater consistency in terminology, particularly around "experts," "stakeholders," "participants," and "carers." Defining these clearly once, and then using the terms consistently, will improve clarity.

• If relevant, it may be useful to reference intervention development frameworks (e.g., TIDieR, GUIDED, BCW/COM-B) to help structure the description of the development process and enhance rigor.

• Consider whether a diagram or table summarising the phases of development, stakeholder groups, and changes made at each step would improve readability and transparency.

Abstract

• Line 31: The study design should be described as the development of a carer program.

Introduction

• You use the term "culturally tailored" (Line 77), but it's not yet clear how this is being defined. I recommend drawing on the cultural adaptation literature, which distinguishes between surface-level and deep-structure adaptations. As the central aim is to develop a culturally tailored program, it would be helpful to clearly define these concepts and explain how your program is culturally relevant.

• Lines 81–88: This content fits better in the Methods section as part of the intervention development process. The Introduction would be strengthened by instead outlining existing programs/interventions in this space and establishing the specific need for this program.

Methods

• Overall, the flow of this section is a little difficult to follow, as the sequence of development is not clearly outlined. Consider restructuring to follow the logical process (e.g., Phase One, recruitment, consultation process, program adjustments etc.).

• Additional detail is needed on how the program was culturally tailored.

• You refer to "experts" several times. For clarity, consider adding a brief statement the first time you use this term—for example, how "experts" were defined and which professions were included.

• Line 110: Please indicate how many phases were included in the development.

• Lines 124–128: The rationale for culturally adapting the package could be strengthened. Providing clear definitions and more detail in the Introduction will help justify this.

• Lines 134–136: Please specify the numbers within each profession (e.g., neurologist [n=1], physiotherapists [n=3], etc.).

• Line 137: Please indicate how many carers were engaged.

• Line 140: More detail is needed about the logistics—how many meetings were held, duration, format (online/in-person), frequency, etc. As this is a development paper, this level of detail is essential.

• Line 159: This sentence repeats professions already listed. Consider defining your "steering committee" or "stakeholder group" early in the Methods and using that term consistently.

• Lines 161–162: If "eligible participants" refers to carers, please clarify this (e.g., "eligible participants (i.e., carers)").

• Line 192: As above, please clarify who "participants" refers to.

• Line 213: How many carers contributed to the validation process?

Results

• It is often unclear who is being referred to as "experts" (e.g., Lines 240, 267). More specificity here will improve clarity.

• Lines 289–290: There is evidence that carers can become overwhelmed with excessive information. Did carers have options to select content relevant to them? Were there any progression controls (e.g., needing to complete one week before the next is unlocked)? How long is each module? Can modules be paused or resumed?

• Since the Introduction highlighted a gap in culturally relevant programs, it would be important to show in the Results how you evaluated whether the resulting program met this aim.

Discussion

• Lines 302–303: You note that the CEP was “tailored for caregivers,” but it remains unclear how this tailoring was achieved. Was this individual tailoring or cultural tailoring? If culturally, how so? Translation alone is only one aspect of cultural adaptation.

Reviewer **3**

Name **Dsouza, Sebestina**

Affiliation **Manipal Academy of Higher Education, Occupational Therapy**

Date **02-Dec-2025**

COI **None**

The study addresses an important gap in stroke rehabilitation. However, there are several methodological concerns that need clarification.

1. Would one year experience be adequate to describe as an 'expert'? This needs some justification as one year could also be considered as inadequate for developing expertise. It may help to add a table on the characteristics of the experts (with area and years of experience) and caregivers

2. The participants were given just 3 days to review the material which may be inadequate and may have caused a rushed review and thus the high scores.

3. A primary aim of the developed material was to make it accessible. So how was the language demands addressed for people with limited literacy? Was the readability analysed? How were translations managed and evaluated?

4. The scope of application of the developed material needs to be clear. Is it for acute stroke or chronic stroke? The content appears to be for acute and bed ridden stroke survivors.

5. It would be good to provide details of how the qualitative feedback was analysed. Currently, it only states that open ended comments were collected.

6. It would be good to have transparency with respect to the development of the material, at least initial version and final version and how feedback was incorporated in the refinement. Also, some feedback may have been difficult to incorporate. How do the authors propose to address the same?

7. The introduction could have a section on review of existing material (manuals/apps) for stroke survivors and caregivers. There are several such material developed by International stoke organizations. So what gap does this material fill? It could help justify the need for developing this material. If I understand correctly, the major aspect of the developed material is cultural relevance. But besides a brief mention in the introduction and a mention of language in the methods, cultural relevance is not highlighted throughout the study and in the content of the developed material either. The content looks similar to other

educational interventions/apps. The introduction should also emphasize more on the impact of stroke in Malaysia with statistics and clarify how the developed material would reduce

8. The discussion could also compare the developed apps/material with the existing ones in Malaysia that are briefly mentioned in the introduction.

9. The last paragraph of the discussion (367-378) gives the impression that the authors are going far away from the study findings. It would be better to restrict the implications to the study findings. Before RCTs, a basic pilot or feasibility study may be required

10. The authors could be cautious about commenting on the application of the developed material in LMICs where access to internet and android phone is itself a challenge, compounded by low literacy and language barriers to use of printed material. There are existing apps developed for LMICs that have not gone beyond research. It may be better to restrict the scope to Malaysia.

VERSION 1 - AUTHOR RESPONSE

Reviewer 1 – Dr. Amelia Adcock

Comment 1

A potential pitfall is the small face validation including only 14 informal caregivers.

Response: We acknowledge this limitation and have discussed it explicitly (Discussion (Line 518-520))

Comment 2

It is unclear how many of the 14 participants interacted with the app and provided feedback.

Response:We have clarified that all 14 caregivers interacted with both the mobile application and the printed guidebook prior to completing face validation (Results : Face Validation section (Line 353-356))

Comment 3

Post-stroke depression should be added under “stroke complications and prevention” rather than only mental health.

Response:We agree with the importance of post-stroke depression. Due to feasibility considerations in the current development phase, this content was included within the

mental health module. We have now explicitly acknowledged this and outlined plans for future integration (Discussion (Line 479-482)

Comment 4

Please explicitly state what resources are available for technological queries versus professional healthcare support.

Response:We have clarified and differentiated technical support pathways and healthcare/professional support resources (Discussion (Line 440-445))

Reviewer 2 – Dr. Melissa Jammal

General Comment 1

Greater consistency in terminology (experts, stakeholders, participants, carers) is needed.

Response:We have defined all key terms at first use and applied consistent terminology throughout the manuscript (Method (Line 113-120)

General Comment 2

Reference intervention development frameworks to strengthen rigor.

Response: In this study , we used The ADDIE instructional design framework. We explained more details and consistently referenced throughout the Methods (Line 127-129, Line 135-173) and Discussion sections (Line 397-398)

Methods-related Comments (flow, phases, numbers, logistics)

Response: We restructured the Methods section to clearly reflect

Phase 1 (needs assessment) :Line 135-142

Phase 2 (design, development, validation) Line :143 - 150

Numbers by profession and caregivers : Line 155-158

Meeting format, duration, and frequency: Line 160-167

Results Comment

Clarify whether caregivers could control content pacing and avoid information overload.

Response:We clarified that the CEP is designed for self-paced, non-sequential use with pause and replay options with additional details on the module (Results : Line 289- 299)

Discussion Comment

Clarify whether tailoring was individual or cultural.

Response: We explicitly clarified that the CEP involved cultural tailoring (primarily surface-structure) rather than individual-level tailoring (Discussion Line 473-490)

Reviewer 3 – Prof. Sebestina Dsouza

Comment 1

Is one year of experience sufficient to define an expert?

Response: We clarified and justified the operational definition of "expert" based on clinical role, active practice, and formal stroke training.(Methods : Lien 113-118)

Comment 2

Three days may be insufficient for review.

Response:We justified this timeframe pragmatically and acknowledged it as a limitation (Methods → Content Validation (Lie 196-201) and Face Validation sections(Line 225-230), Discussion → Limitations paragraph (Line 522-526)

Comment 3

How was accessibility and literacy addressed?

Response:We described plain-language development, infographic use, translation procedures, and acknowledged the lack of formal readability testing (Results → Module Development Line 273- 276) , Discussion → Limitations paragraph Line 520-522)

Comment 4

Clarify scope: acute vs chronic stroke.

Response: We clarified that the CEP targets early post-acute and community care, particularly for survivors with mobility limitations or bed-bound status (Methods → Package Development : Line 129-132)

Comment 5

Clarify how qualitative feedback was analysed.

Response:We clarified that qualitative comments were analysed descriptively to inform refinement rather than thematic theory-building (Methods : Line 241-246)

Comments 6–10 (transparency, gap justification, overreach, LMIC caution)

Response: We

Clearly described changes from draft to final version (Result: Line 327-351,page 20)

Strengthened justification of the Malaysian gap (Introduction: Line 92-99,Discussion: 387-393)

Restricted implications to Malaysia (Discussion : Line 492-493)

Positioned pilot/feasibility studies before RCTs (Discussion : Line 509-512)

We believe that all reviewer and editor comments have been fully addressed and that the revised manuscript is now clearer, more transparent, and methodologically robust. We sincerely appreciate the reviewers' guidance and hope that the revised manuscript meets the journal's expectations.
